# Diversity of *Micrurus* Snake Species Related to Their Venom Toxic Effects and the Prospective of Antivenom Neutralization

**DOI:** 10.1371/journal.pntd.0000622

**Published:** 2010-03-09

**Authors:** Gabriela D. Tanaka, Maria de Fátima D. Furtado, Fernanda C. V. Portaro, Osvaldo Augusto Sant'Anna, Denise V. Tambourgi

**Affiliations:** 1 Immunochemistry Laboratory, Butantan Institute, São Paulo, SP, Brazil; 2 Herpetology Laboratory, Butantan Institute, São Paulo, SP, Brazil; University of Kelaniya, Ragama, Sri Lanka

## Abstract

**Background:**

*Micrurus* snake bites can cause death by muscle paralysis and respiratory arrest, few hours after envenomation. The specific treatment for coral snake envenomation is the intravenous application of heterologous antivenom and, in Brazil, it is produced by horse immunization with a mixture of *M. corallinus* and *M. frontalis* venoms, snakes that inhabit the South and Southeastern regions of the country. However, this antivenom might be inefficient, considering the existence of intra- and inter-specific variations in the composition of the venoms. Therefore, the aim of the present study was to investigate the toxic properties of venoms from nine species of *Micrurus*: eight present in different geographic regions of Brazil (*M. frontalis, M. corallinus, M. hemprichii, M. spixii, M. altirostris, M. surinamensis, M. ibiboboca, M. lemniscatus*) and one (*M. fulvius*) with large distribution in Southeastern United States and Mexico. This study also analyzed the antigenic cross-reactivity and the neutralizing potential of the Brazilian coral snake antivenom against these *Micrurus* venoms.

**Methodology/Principal Findings:**

Analysis of protein composition and toxicity revealed a large diversity of venoms from the nine *Micrurus* species. ELISA and Western blot assays showed a varied capability of the therapeutic antivenom to recognize the diverse species venom components. *In vivo* and *in vitro* neutralization assays indicated that the antivenom is not able to fully neutralize the toxic activities of all venoms.

**Conclusion:**

These results indicate the existence of a large range of both qualitative and quantitative variations in *Micrurus* venoms, probably reflecting the adaptation of the snakes from this genus to vastly dissimilar habitats. The data also show that the antivenom used for human therapy in Brazil is not fully able to neutralize the main toxic activities present in the venoms from all *Micrurus* species occurring in the country. It suggests that modifications in the immunization scheme, with the inclusion of other venoms in the antigenic mixture, should occur in order to generate effective therapeutic coral snake antivenom.

## Author Summary

The Elapidae family is represented in America by three genera of coral snakes: *Micruroides*, *Leptomicrurus* and *Micrurus*, the latter being the most abundant and diversified group. *Micrurus* bites can cause death by muscle paralysis and respiratory arrest few hours after envenomation. The specific treatment for *Micrurus* envenomation is the application of heterologous antivenom. The aim of this study was to compare the toxicity of venoms from nine species of coral snakes and analyze the neutralization potential of the Brazilian coral snake antivenom. *In vitro* assays showed that the majority of the *Micrurus* venoms are endowed with phospholipase and hyaluronidase and low proteolytic activities. These enzymes are not equally neutralized in all venoms by the therapeutic antivenom. Moreover, *in vivo* assays showed that some of the *Micrurus* venoms are extremely lethal, such as the ones from *M. altirostris*, *M. corallinus*, *M. frontalis*, *M. lemniscatus* and *M. spixii*. Neutralization tests, performed *in vivo*, showed that the therapeutic antivenom was able to neutralize better the venoms from *M. frontalis*, *M. corallinus*, and *M. spixii* but not from *M. altirostris* and *M. lemniscatus*. Taken together, these results suggest that modifications in the immunization antigenic mixture should occur in order to generate more comprehensive therapeutic antivenom.

## Introduction

The Elapidae family has about 250 species, distributed from the Southeastern and Southwestern United States, through Mexico, Central America and South America, and are also found in Asia, Africa and Australia [1]. In the Americas, there is a group of more than 120 species and subspecies, divided into three genera: *Micruroides*, with one species; *Leptomicrurus* with three, and *Micrurus*, with almost 70 species [1],[2],[3],[4].

Coral snakes have a large geographical distribution in Americas, inhabiting extremely diverse environments, from lowland rainforests and deserts to highland cloudy forests [1],[5]. Most of the snakes of the *Micrurus* genus has terrestrial to subfossorial habits, however, some species are semi-aquatic, such as *M. surinamensis* and *M. lemniscatus* [1].

Most coral snakes have a color pattern of some combination of the red, yellow or white, and black, usually disposed in rings. They are proterogliphous animals, presenting the fixed small teeth at the forefront of the mouth. Food is generally composed of small snakes, but may also include lizards and amphisbaenians. Certain species have specialized nutritional habits, feeding on caecilians, swamp eels, and other type of fishes and even onycophorans and other invertebrates [1]. Snakes such as *M. lemniscatus* and *M. surinamensis* feed on fish and *M. hemprichii* of peripatus [6],[7],[5].

The *Micrurus* species of public health importance are *M. fulvius* in the United States and Mexico, *M. alleni*, *M. diastema* and *M. nigrocintus* in Central America and *M. altirostris*, *M. corallinus*, *M. dumerilii*, *M. frontalis*, *M. mipartitus*, *M. spixii*, *M. surinamensis* and *M. isozonus* in South America [8],[9],[10]. In Brazil, some species are quite common and widespread in large areas of the territory, such as *M. corallinus*, *M. frontalis*, *M. ibiboboca*, *M. lemniscastus*, *M. spixii* *and M. surinamensis*.

Human envenomations by coral snakes are relatively rare due to their subfossorial habits; however, the case fatality, attributable to respiratory paralysis, may be high [11]. A variety of local and systemic manifestations of envenoming has been described in patients bitten by different species of coral snakes [5],[12],[11]. The main feature of the venom action is the neurotoxicity, although, experimentally, it has been reported that some *Micrurus* venoms may induce myotoxicity and local lesions [13],[14]. Neurotoxicity can be produced by a post-synaptic action, through blockage of the end-plate receptors by alpha neurotoxins, as determined for *M. frontalis* venom, or by a pre-synaptic-like activity, which causes inhibition of acetylcholine release at the motor nerve endings, as induced by *M. corallinus* venom. There are also venom toxins, such as cardiotoxins and myotoxic phospholipases A_2_ from *M. nigrocinctus* and *M. fulvius*, which block the end-plate receptors and depolarize the muscle fiber membrane [15].

Experimental studies have shown that *Micrurus* venoms are cardiotoxic, myotoxic, hemolytic, hemorrhagic and edematogenic [13],[14],[16],[17],[18],[19],[20],[21],[22],[23],[24]. Furthermore, many enzymatic activities have been detected including those derived from phospholipase A_2_, hyaluronidase, phosphodiesterase, leucine amino peptidase, L-amino acid dehydrogenase, acetylcholinesterase, alkaline phosphomonoesterase and L-amino acid oxidase actions [25],[26]. Anticoagulant action was also identified in some coral venom species, but none or little proteolytic activity was detected [26]. Therefore, common characteristics, as well as variability of some biological properties, have been demonstrated in comparative studies of *Micrurus* venoms [23],[25],[26],[27],[28].

The transcriptomic analysis of a *Micrurus* snake venom gland (*M. corallinus*) was recently described [29]. Toxin transcripts represented 46% of the total ESTs and the main toxin classes were neurotoxins, *i.e*, three-finger toxins (3FTx) and phospholipases A_2_ (PLA_2_s). It was also showed that the post-synaptic components (3FTx) were very diverse in terms of sequences, possibly aiming to achieve different types of receptors, whereas the pre-synaptic component (PLA_2_) was more conserved. The high expression of both types of these neurotoxins is in agreement with the known presence of pre- and post-synaptic activities in the *Micrurus* venoms. However, eight other classes of toxins were found, including C-type lectins, natriuretic peptide precursors and high-molecular mass components such as metalloproteases and L-amino acid oxidases.

The specific treatment for *Micrurus* envenomation is the intravenous application of heterologous antivenom. In Brazil, the coral snake therapeutic antivenom produced by Butantan Institute is obtained by the immunization of horses with a mixture containing equivalent amounts of *M. corallinus* and *M. frontalis* venoms [30]. In view of the fact that *Micrurus* venoms can exhibit a diversity of composition and toxicity, the therapeutic antivenom may not be capable to fully recognize all the major components of the distinct venom species occurring in the country. Therefore, the aim of this study was to characterize some biological properties of venoms from nine species of *Micrurus*, including those used for serum preparation, *i.e*., *M. frontalis* and *M. corallinus*, evaluate their antigenic cross-reactivity, using the Brazilian coral snake antivenom, as well as to test the ability of this antivenom to neutralize the main toxic activities of these venoms.

## Materials and Methods

### Chemicals and reagents

Triton X-100, Tween 20, bovine serum albumin (BSA), L-α-phosphatidylcholine, ortho-phenylenediamine (OPD), hyaluronic acid and goat anti-horse (GAH) IgG horseradish peroxidase labeled (IgG-HRPO) were purchased from Sigma (St. Louis, Missouri, USA). Goat anti-horse (GAH) IgG labeled with alkaline phosphatase (IgG-AP), 5-bromo-4-chloro-3-indolyl-phosphate (BCIP) and nitroblue tetrazolium (NBT) were from Promega Corp. (Madison, Wisconsin, USA). The P2E Fluorescent Resonance Energy Transfer (Abz-FEPFRQ-EDnp) substrate was synthesized and purified as described in Hirata et al. [31].

### Venoms

Venoms from *Micrurus frontalis*, *M. corallinus*, *M. ibiboboca*, *M. hemprichii*, *M. spixii*, *M. fulvius*, *M. altirostris*, *M. surinamensis* and *M. lemniscatus* were supplied by Herpetology Laboratory from Butantan Institute, SP, Brazil. Stock solutions were prepared in PBS (10 mM sodium phosphate containing 150 mM NaCl, pH 7.2) at 1.0 mg/mL.

### Coral snake antivenom

The Brazilian therapeutic coral snake antivenom, produced by hyperimmunization of horses with venoms from *M. corallinus* (50%) and *M. frontalis* (50%), was obtained from Butantan Institute, SP, Brazil.

### Electrophoresis and western blot

Samples of 20 µg of *Micrurus* venoms were solubilised in non-reducing sample buffer and run on 7.5 to 15% SDS-PAGE gradient gels [32]. Gels were stained with silver [33] or blotted onto nitrocellulose [34]. After transfering, the membrane was blocked with PBS containing 5% BSA and incubated with the coral snake antivenom (diluted 1∶2,000) for 1 h at room temperature. The membrane was washed 3 times for 10 min with PBS/0.05% Tween 20, and incubated with GAH/IgG-AP (1∶7,500) in PBS/1% BSA for 1 h at room temperature. After washing 3 times for 10 min with PBS/0.05% Tween 20, the blot was developed using NBT/BCIP according to the manufacturer's instructions (Promega).

### Determination of LD_50_

The lethal potential of *Micrurus* venoms was assessed in Swiss mice by intraperitoneal injection of different amounts of venoms in 500 µL of PBS. Four animals were used for each venom dose (five doses). The LD_50_ was calculated by probit analysis of death occurring within 48 h of venom injection [35]. All animal experiments were approved in advance by the Laboratory Animal Ethics Committee of Butantan Institute.

### Phospholipase activity

The phospholipase A_2_ activity of *Micrurus* venoms was determined as described by Price III [36], with some modifications. Samples of the venoms (4 µg) and PBS were added to a final volume of 200 µL. Samples of 180 µL of the mixture containing: 5 mM Triton X-100, 5 mM phosphatidylcholine (Sigma), 2 mM HEPES, 10 mM calcium chloride and 0,124% (wt/vol) bromothymol blue dye in water, at pH 7.5 and at 37°C, were added. After a pre-incubation of 5 min at 37°C, the absorbance of the samples was determined at λ 620 nm in a Multiskan spectrophotometer EX (Labsystems, Finland). Results were expressed in nanomoles of acid *per* minute *per* µg of venom (compared on pH changes in standard curves of the reaction mixture using HCl).

### Proteolytic activity

Samples of the *Micrurus* venoms (50 µg) were mixed with 5 µM of the Fluorescent Resonance Energy Transfer (FRET) substrate, Abz-FEPFRQ-EDnp, and PBS, for a final volume of 100 µL, and the reactions monitored by measuring the fluorescence (λ_em_ 420 nm and λ_ex_ 320 nm) in a spectrofluorimeter (Victor 3™, Perkin-Elmer, USA) at 37°C, as described by Araújo et al. [37]. The specific proteolytic activity was expressed as units of free fluorescence *per* minute *per* µg of venom (UF/min/µg).

### Hyaluronidase activity

Hyaluronidase activity was measured as described [38], with slight modifications. Samples of *Micrurus* venoms (30 µg) were added to 100 µL of the hyaluronic acid substrate (1 mg/mL) and acetate buffer (pH 6.0) for a final volume of 500 µL. The mixtures were incubated for 15 min at 37°C. After the incubation, it was added to the samples 1 mL of cetyltrimethylammonium bromide 2.5% in NaOH 2%, to develop the turbidity in the mixtures, and the absorbance measured in a spectrophotometer (Multiskan EX) at λ_em_ 405 nm. Results were expressed in units of turbidity reduction (UTR) *per* mg of venom.

### Enzyme linked immunosorbent assay (ELISA)

Microtitre plates were coated with 100 µL of *Micrurus* venoms (10 µg/mL; overnight at 4°C). Plates were blocked with 5% BSA in PBS and increased dilutions of the therapeutic coral snake antivenom were added. After 1 h of incubation at room temperature, plates were washed with PBS/0.05% Tween 20 and incubated with GAH-IgG-HRPO diluted 1∶3,000, for 1 h at room temperature. Plates were washed and the reactions developed with OPD substrate according to the manufacturers conditions (Sigma). The absorbances were recorded in an ELISA reader (Multiskan spectrophotometer EX) at λ 492 nm. The titer was established as the highest antivenom dilution, in which an absorbance five times greater than that determined for the normal horse serum was measured.

### Serum neutralization assays performed *in vitro*

The ability of the therapeutic Brazilian coral snake antivenom to neutralize the venoms phospholipase, hyaluronidase and proteolytic activities was estimated by incubating *Micrurus* venoms with the antivenom. The antivenom volume, amount of venoms and the pre-incubation time, for each tested enzymatic activity, was standardized using the immunization pool, composed by 50% of *M. corallinus* and 50% of *M. frontalis* venoms. For serum neutralization measurements of the phospholipase activity, samples of 4 µg of the venoms were incubated with the antivenom, diluted 1∶10; for the hyaluronidase activity, samples of *Micrurus* venoms (30 µg) and the antivenom (1∶20) were incubated for 20 min at room temperature; for the proteolytic activity, samples of *Micrurus* venoms (50 µg) and the antivenom (1∶4) were incubated for 10 min at room temperature. Venoms residual toxic activities were measured as described above.

### Neutralization of the lethal activity

The capacity of the therapeutic coral snake antivenom to neutralize the lethal activity of *Micrurus* venoms was determined by mixing the venoms, corresponding to 2 LD_50_, with serial dilutions of the horse antivenom. The mixtures were incubated for 30 min at 37°C and the animals received 0.5 mL by the intraperitoneal route. The effective dose (ED_50_) was calculated from the number of deaths within 48 h of injection of the venom/antivenom mixture using probit analysis, as described above. The ED_50_ was expressed as mL of antivenom *per* µg of venom.

## Results

### Eletrophoretic characterization of *Micrurus* venoms

**Figure 1:**
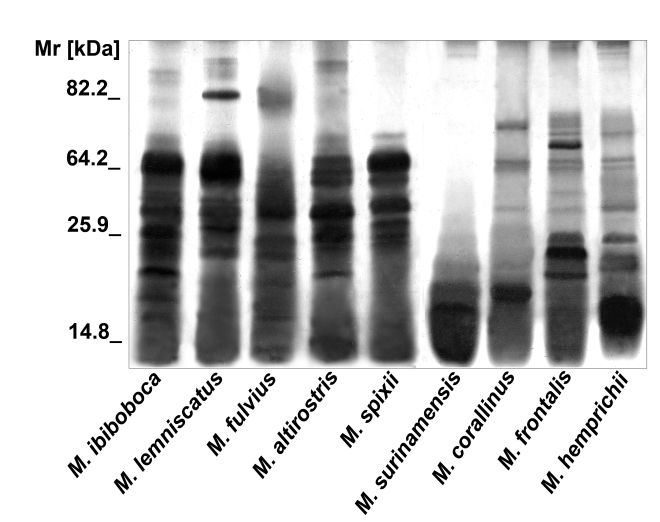
SDS-polyacrylamide gel eletrophoresis. Samples (20 µg) of *M. ibiboboca*, *M. lemniscatus*, *M. fulvius*, *M. altirostris*, *M. spixii*, *M. surinamensis*, *M. corallinus*, *M. frontalis* and *M. hemprichii* venoms were analyzed by SDS-PAGE in a gradient gel (7.5% to 15%) and silver stained.

The protein profiles of *Micrurus* venoms were analyzed by SDS-PAGE followed by silver staining. **Figure 1** shows that the venoms from the nine coral species differ in composition, number and intensity of bands. The majority of the components of these venoms present Mr inferior to 64 kDa. Venom from *M. surinamensis* differs more from the others, by the presence of a few number of components with Mr lower than 20 kDa.

### Enzymatic activities of the *Micrurus* venoms

In order to assess whether the venoms of *Micrurus* displayed the same biological activities, some functional assays were carried out.

The LD_50_, used as a parameter of the *Micrurus* venom neurotoxicity, was tested in groups of mice, after intraperitoneal injection of different concentrations of the venoms, and the number of deaths recorded during 48 h. The LD_50_ values, calculated by probit analysis at 95% confidence, were variable among *Micrurus* venoms, being the most lethal those from *M. lemniscatus, M. altirostris*, *M. spixii*, *M. corallinus* and *M. frontalis* **(** **Table 1** **)**.

**Table 1:** Lethal dose 50% (LD_50_) of *Micrurus* spp snake venoms determined in murine model.

Venoms	LD_50_ (µg)
***Micrurus ibiboboca***	76 (67–89)
***Micrurus lemniscatus***	13 (7–22)
***Micrurus fulvius***	64 (52–88)
***Micrurus altirostris***	9 (7–13)
***Micrurus spixii***	8 (6–16)
***Micrurus surinamensis***	58 (43–87)
***Micrurus corallinus***	7 (5–27)
***Micrurus frontalis***	22 (4–29)
***Micrurus hemprichii***	47 (20–88)

Results are expressed in µg venom/mouse (18–22 g), and the 95% confidence limits are included in parenthesis.

**Figure 2:**
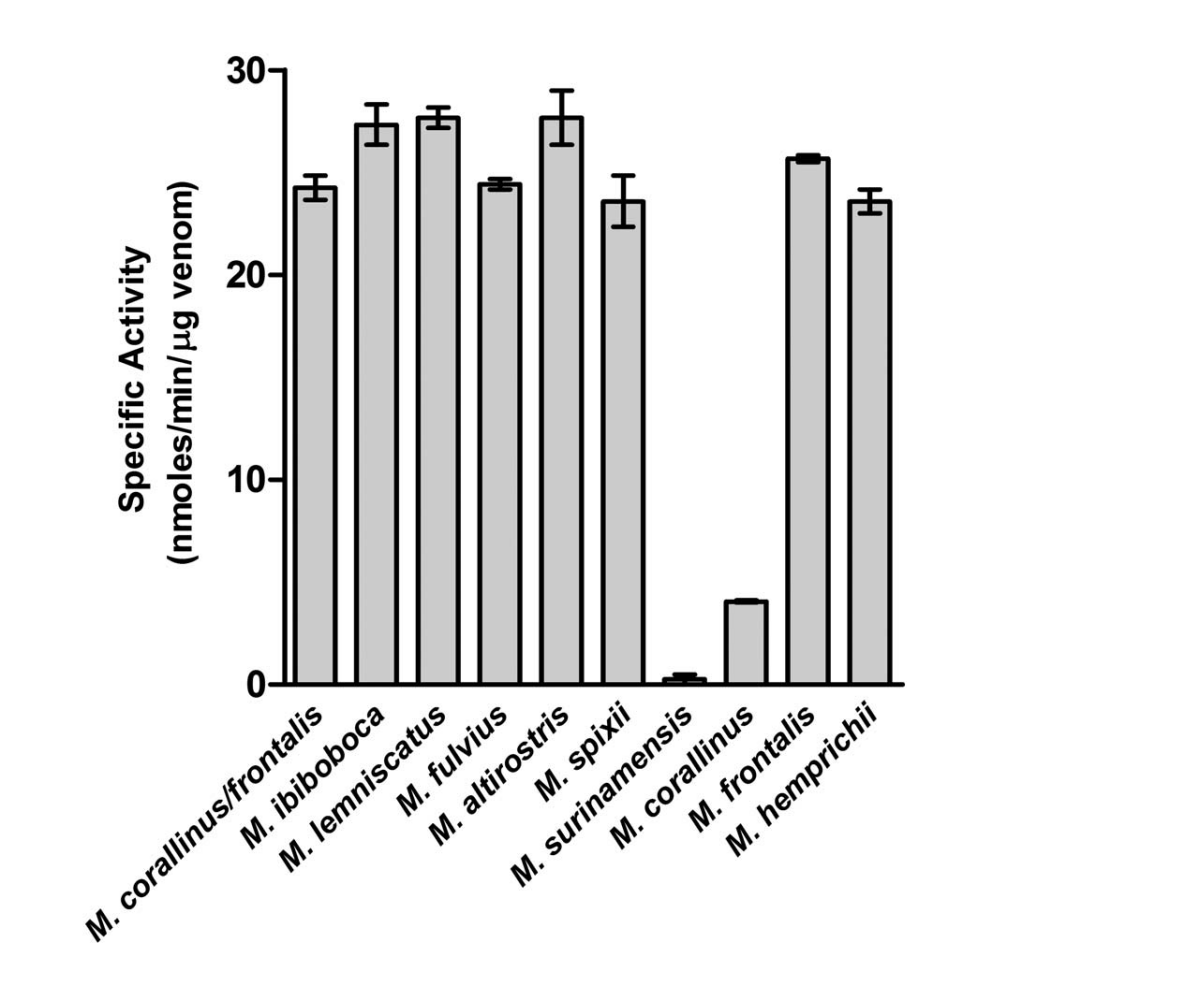
Determination of the phospholipase A_2_ activity. Samples of individual *Micrurus* venoms (4 µg), or a mixture of *M. frontalis* (2 µg) and *M. corallinus* (2 µg), venoms were incubated for 20 min at 37°C with 180 µL of a mixture containing 5 mM Triton X-100, 5 mM phosphatidylcholine, 2 mM HEPES, 10 mM calcium chloride and 0.124% (wt./vol) bromothymol blue dye in water. Results are representative for three separate experiments and expressed as nanomoles acid *per* minute *per* µg of venom.

**Figure 2** shows that venoms contain variable levels of PLA_2_ activity, toxin also associated with *Micrurus* envenomation neurotoxicity. *M. ibiboboca, M. lemniscatus, M. fulvius, M. altirostris, M. spixii, M. frontalis, M. hemprichii* and the mixture of *M. corallinus* and *M. frontalis* venoms present an intense hydrolytic activity. In the same experimental conditions, the venoms from *M. corallinus* and *M. surinamensis* showed, respectively, low and none phospholipase activity.

**Figure 3:**
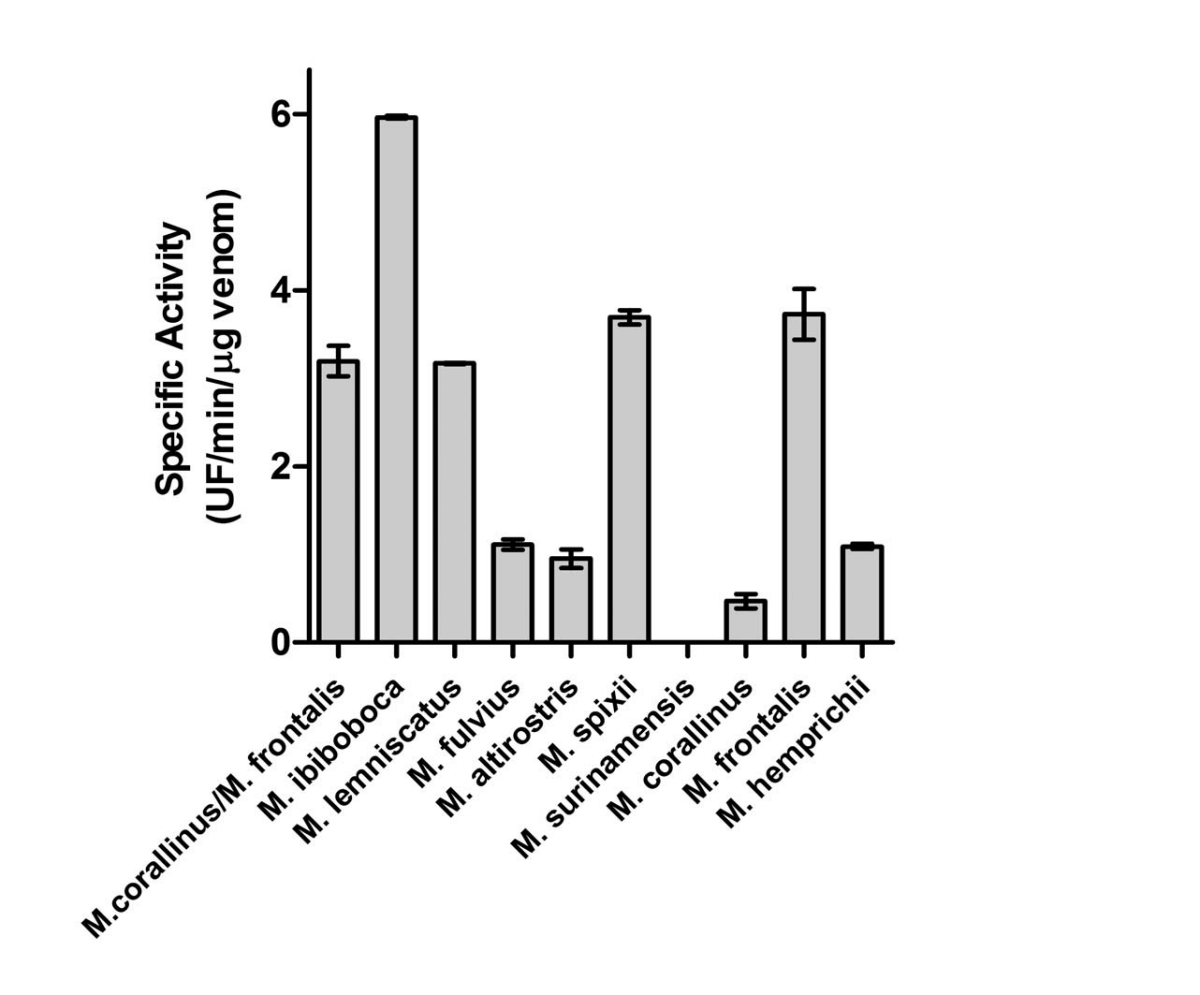
Determination of the proteolytic activity. Samples of *Micrurus* venoms (50 µg), or a mixture of *M. frontalis* (25 µg) and *M. corallinus* (25 µg), were incubated at 37°C with the FRET substrate, Abz-FEPFRQ-EDnp, and the hydrolysis measured in a spectrofluorimeter. Results are representative for three separate experiments and expressed as units of free fluorescence *per* minute *per* µg of venom (UF/min/µg).

The proteolytic activity of the *Micrurus* venoms was tested using a FRET substrate, Abz-FEPFRQ-EDnp. **Figure 3** demonstrates that the venoms from *M. ibiboboca, M. lemniscatus, M. fulvius, M. altirostris, M. spixii, M. corallinus, M. frontalis and M. hemprichii* present hydrolytic activity on this substrate. However, no proteolytic could be measured in the venom from *M. surinamensis*.

**Figure 4:**
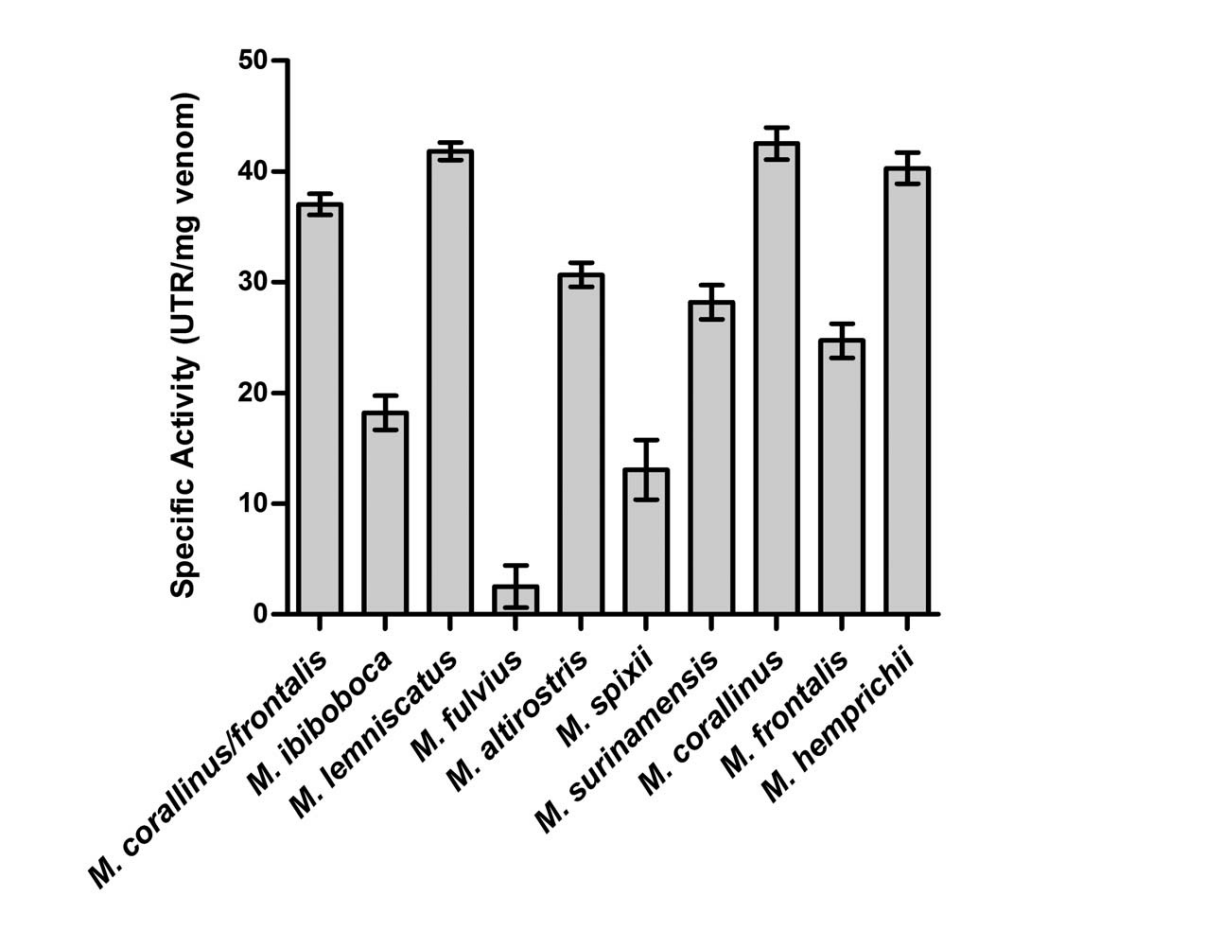
Determination of the hyaluronidase activity. Samples of *Micrurus* venoms (30 µg), or a mixture of *M. frontalis* (15 µg) and *M. corallinus* (15 µg), were incubated for 15 min at 37°C with the hyaluronic acid as substrate. After this period, it was added cetyltrimethylammonium bromide, to develop the turbidity in the mixtures, and the absorbance measured in a spectrophotometer at λ_em_ 405 nm. Results are representative for three separate experiments and expressed in units of turbidity reduction (UTR) *per* mg of venom.

The hyaluronidase activity was also analyzed and **Figure 4** illustrates that it is high in the venoms of *M. lemniscatus*, *M. corallinus*, *M. hemprichii* and mixture of *M. corallinus* and *M. frontalis*. *M. ibiboboca, M. altirostris*, *M. surinamensis*, *M. frontalis* and *M. spixii* displayed intermediate activity followed by *M. fulvius* venom, which presents low hyaluronidase activity.

### Immunochemical cross-reactivity

**Figure 5:**
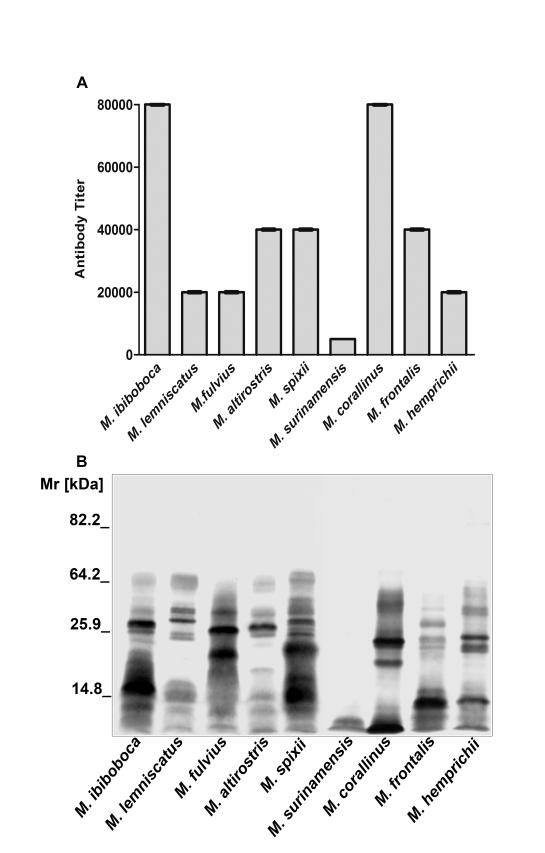
Cross-reactivity of coral snake antivenom. [**A**] ELISA: plates were coated with 10 µg of *Micrurus* venoms and incubated with different dilutions of coral snake antivenom, followed by GAH/IgG-HRPO, diluted 1∶3,000. The absorbance of the samples was determined at 492 nm. The data presented correspond to the mean OD_492_ value +/− SD of experiments carried out in duplicate. [**B**] Western blot: Samples (20 µg) of *M. ibiboboca*, *M. lemniscatus*, *M. fulvius*, *M. altirostris*, *M. spixii*, *M. surinamensis*, *M. corallinus*, *M. frontalis* and *M. hemprichii* venoms were separated by SDS-PAGE in gradient gel (7.5% to 15%), electrotransfered to a nitrocellulose membrane and incubated with the coral snake antivenom diluted 1∶2,000 followed by GAH/IgG-AP. The reaction was revealed with NBT and BCIP.

The coral snake antivenom, produced by Butantan Institute and used in Brazil for human serum therapy, is obtained by the immunization of horses with a mixture of *M. corallinus* and *M. frontalis* venoms. In an ELISA, this antivenom was tested for cross-reactivity, using *Micrurus* spp venoms as antigens. **Figure 5A** shows the antivenom was mostly effective in detecting components of *M. corallinus* and *M. ibiboboca* venoms. This antivenom presented intermediary titers for *M. lemniscatus, M. fulvius*, *M. altirostris, M. frontalis, M. spixii* and *M. hemprichii* venoms, being the one from *M. surinamensis* poorly recognized.

By western blotting, it was demonstrated that the coral snake antivenom could recognize several but not all components present in the *Micrurus* spp venoms. *M. surinamensis* venom components were weakly detected. The antivenom was also unable to recognize molecules with Mr above 64 kDa present in the majority of the venoms **(** **Fig. 5B** **)**.

### *In vitro* antivenom neutralization assays

**Figure 6:**
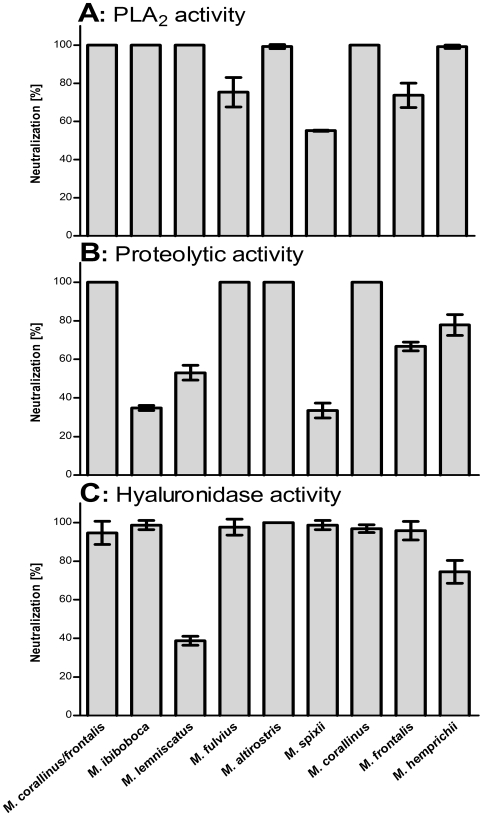
*In vitro* serum neutralization assays. [**A**] Phospholipase A_2_ activity: Samples of the venoms (4 µg) were incubated with the coral snake antivenom (1∶10) for 20 min at room temperature. As positive control, the mixture of *M. corallinus* (2 µg) and *M. frontalis* (2 µg) venoms, used for the production of the antivenom, was used. [**B**] Proteolytic activity: Samples of *Micrurus* venoms (50 µg) and the coral snake antivenom (1∶4) were incubated for 10 min at room temperature. As positive control, the mixture of the *M. corallinus* (25 µg) and *M. frontalis* (25 µg) venoms was used. [**C**] Hyaluronidase activity: Samples of *Micrurus* venoms (30 µg) and the coral snake antivenom (1∶20) were incubated for 15 min at room temperature. As positive control, the mixture of the *M. corallinus* (15 µg) and *M. frontalis* (15 µg) venoms was used. Venoms residual toxic activities were measured as described in materials and methods. Results are representative for three separate experiments and expressed as percentage of neutralization of the venoms enzymatic activities.

In order to analyze if the Brazilian coral snake antivenom could neutralize the enzymatic activities present in the *Micrurus* spp venoms, some *in vitro* assays were performed. **Figure 6A** shows that the antivenom, used in a dilution capable of neutralizing 100% of the phospholipase activity present in the mixture of *M. frontalis* and *M. corallinus* venoms (positive control), was able to completely inhibit this activity in *M. ibiboboca, M. lemniscatus, M. altirostris, M. corallinus* and *M. hemprichii* venoms. However, it could partially neutralize the venoms from *M. fulvius* (57.4%), *M. spixii* (59%) and *M. frontalis* (76.5%). **Figure 6B** showed that the antivenom was also able to fully neutralize the proteolytic activity present in *M. fulvius*, *M. altirostris* and *M. corallinus* venoms. On the other hand, this activity was partially neutralized in the venoms from *M. ibiboboca*, *M. lemniscatus*, *M. spixii*, *M. frontalis* and *M. hemprichii*. The hyaluronidase activity could be totally inhibited by the therapeutic antivenom in most of the *Micrurus* venoms, with the exception of *M. lemniscatus* and *M. hemprichii* **(** **Fig. 6C** **)**.

### Serum neutralization of the lethal activity

**Figure 7:**
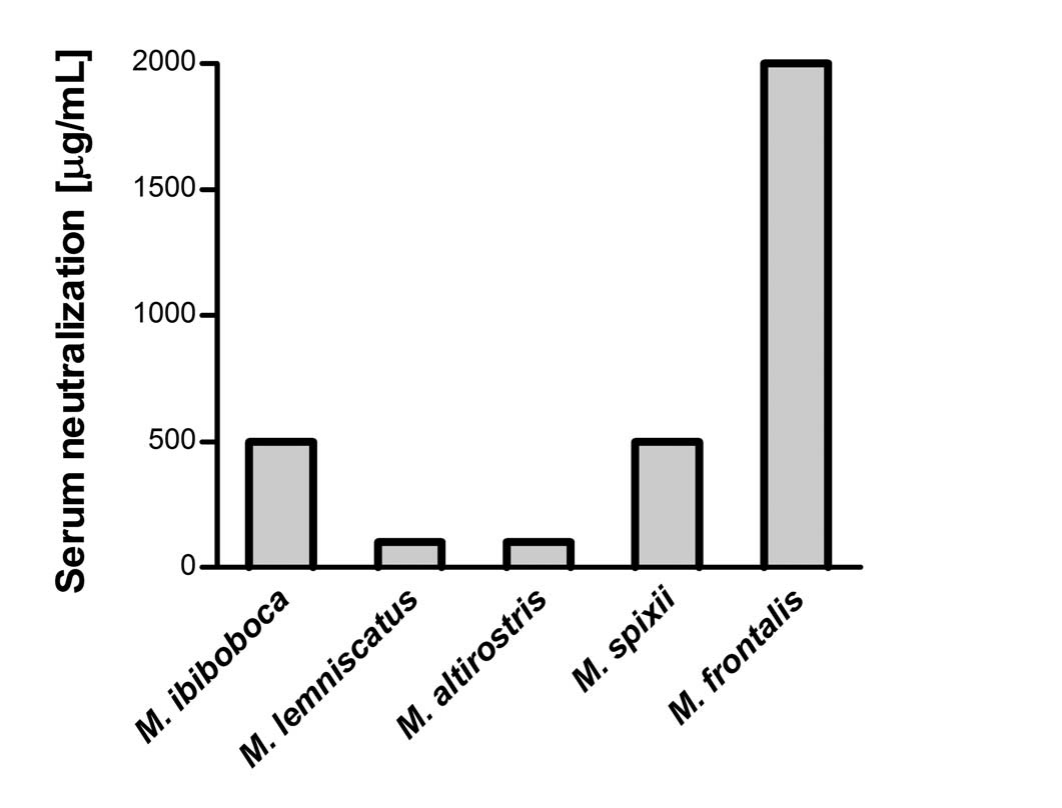
Antivenom neutralization of *Micrurus* venoms lethal toxicity. Samples corresponding to 2 LD_50_, of each *Micrurus* venom, were mixed with serial dilutions of the coral snake horse antivenom. The mixtures were incubated for 30 min at 37°C and the animals were i.p. inoculated. The effective dose (ED_50_) was calculated, from the number of deaths within 48 h of injection of the venom/antivenom mixture, using probit analysis and expressed as mL of antivenom *per* µg of venom.

Some coral snake venoms were chosen for further *in vivo* antivenom neutralization analysis based on their lethal toxicity. **Figure 7** shows that the coral snake antivenom was able to neutralize, although with different potencies, the venoms from *M. spixii*, *M. frontalis and M. corallinus*. However, it could poorly neutralize the venoms from *M. altirostris* and *M. lemniscatus*, with potency values inferior to 100 µg of venom *per* mL of antivenom.

## Discussion

Biochemical studies concerning the *Micrurus* venoms are very scarce, due to difficulties in the correct identification of the species, extraction of the venom and maintenance of the animals in captivity. Previous studies have demonstrated that snakes venoms from *Micrurus* genus present individual variations in composition, related to their geographic distribution, age, gender and diet [22],[39],[40].

In the present study, we have investigated the toxic properties of venoms from nine species of *Micrurus*, the antigenic cross-reactivity and the neutralizing potential of the Brazilian therapeutic coral snake antivenom against these venoms. Results are summarized in **Table 2**.

**Table 2:** Summary of the biological activities of *Micrurus* spp venoms, cross-reactivity and neutralization potential of the Brazilian coral snake antivenom.

Venoms	Biological activities				Antivenom reactivity	Antivenom neutralization			
						*In vitro* test			*In vivo* test
	PLA_2_	P	H	Lethal	Titer (Elisa)	PLA_2_	P	H	Lethal
***M. ibiboboca***	+++	+++	++	+	+++	+++	+	+++	nd
***M. lemniscatus***	+++	++	+++	+++	+	+++	++	+	+/−
***M. fulvius***	+++	+	+	+	+	++	+++	+++	nd
***M. altirostris***	+++	+	++	+++	++	+++	+++	+++	+/−
***M. spixii***	+++	++	++	+++	++	+	+	++	+
***M. surinamensis***	−	−	++	+	+/−	nd	nd	+++	nd
***M. corallinus***	+	+	+++	+++	+++	+++	+++	+++	+
***M. frontalis***	+++	++	++	++	++	++	++	+++	+++
***M. hemprichii***	+++	+	+++	+	+	+++	++	++	nd

**PLA_2_:** Phospholipase A_2_; **P:** Protease; **H:** Hyaluronidase;

High (+++); Intermediate (++); Low (+); Very low (+/−); Absent: (−); not determined (nd).

Analysis of the *Micrurus* spp biological properties, as performed by testing the phospholipase, proteolytic, hyaluronidase and lethal activities showed a great variability in the venoms composition. Thus, data presented here showed that the majority of the venoms present intense PLA_2_ activity, although it is lacking in *M. surinamensis* venom.

Venoms from *Micrurus* genus have been characterized as possessing low or no proteolytic activity [41]. In the present study, using the FRET substrate Abz-FEPFRQ-EDnp, we could identify proteolytic activity in the majority of *Micrurus* venoms, being the exception the *M. surinamensis* venom. Moreover, it was also demonstrated that *Micrurus* venoms posses varied levels of hyaluronidase activity.

The analysis of the lethal potential also showed a large range of toxicity variation among coral snake venoms from *Micrurus* genus. The ones from *M. lemniscatus*, *M. altirostris*, *M. spixii*, *M. corallinus* and *M. frontalis* were the most poisonous. These results are in accordance with those reported by others authors, who studied the lethal toxicities of *M. altirostris* (LD_50_ = 10 µg/kg), *M. spixii* (LD_50_ = 6.7 µg/kg), *M. corallinus* (LD_50_ = 7.1 µg/kg) and *M. frontalis* (LD_50_ = 19.3 µg/kg) [42],[43].

*Micrurus* venom is primarily neurotoxic, causing little local tissue reaction or pain at the bite site. Once clinical signs of coral snake envenomation appear they progress with alarming rapidity and are difficult to reverse. In a recent clinical report, it was observed unusual features of coral snake (*M. lemniscatus helleri*) envenomation, with the patient presenting persistent severe local pain, very slow evolution of neurotoxic envenoming, which after 60 h culminated with respiratory failure [11]. These data reinforce the idea that differences in venoms composition may be responsible for the variety of systemic and local manifestations of coral envenoming.

Our results showed that the Brazilian coral snake antivenom presents a variable capability of recognizing venoms antigens, as demonstrated by differences in the antibody titers, as measured by ELISA, being the lowest the one obtained for *M. surinamensis* venom. By Western blotting, it was revealed that the antivenom recognized components of Mr from 64.2 to 14.8 kDa, presented in the majority of the venoms. In contrast, this antivenom, as also demonstrated by ELISA, weakly recognized proteins from *M. surinamensis* venom.

Some *in vitro* tests were established and performed in order to analyze the neutralization potential of the therapeutic antivenom. It was possible to show that the antivenom was not capable to fully neutralize the phospholipase activity from *M. fulvius, M. spixii and M. frontalis*, the proteolytic activity from *M. ibiboboca*, *M. lemniscatus*, *M. spixii*, *M. frontalis* and *M. hemprichii* and the hyaluronidase activity of the *M. lemniscatus* and *M. hemprichii* venoms. Moreover, neutralization tests performed *in vivo* demonstrated that the coral snake antivenom was capable to neutralize the most lethal *Micrurus* venoms, such as the ones from *M. spixii*, *M. corallinus* and *M. frontalis.* However, this antivenom had low efficacy in neutralizing the high lethal activity of *M. altirostris* and *M. lemniscatus* venoms.

Higashi et al. [43] have demonstrated that anti-*M. corallinus* antivenom was able to neutralize, *in vivo,* the lethal effect of *M. corallinus* venom, but not from *M. frontalis*, *M. ibiboboca* and *M. spixii* venoms. In contrast, antivenom against *M. frontalis* could neutralize the lethal effect of *M. frontalis*, *M. ibiboboca* and *M. spixii* venoms, but not from *M. corallinus*. Abreu et al. [44] showed that the commercial and experimental coral antivenoms have low efficacy in neutralizing the *M. altirostris* venom neurotoxicity as measured in *in vitro* and *in vivo* (inhibition of the lethality) assays.

Table 2 shows that the antivenom antibody titers had no positive correlation with its neutralization potential, indicating that *in vitro* and *in vivo* neutralization tests are fundamental to determine the efficacy of the therapeutic antivenom.

Multivalent coral snake antivenom has been also prepared, in horses, against a mixture of venoms from *M. nigrocinctus*, *M. mipartitus* and *M. frontalis* species [45]. In this study it was suggested that it would be useful in treating bites from most of the important coral snake species in North and South America, such as *M. fulvius*, *M. alleni*, *M. carinicaudus dumerilii*, *M. corallinus*, *M. frontalis*, *M. lemniscatus*, *M. mipartitus*, *M. nigrocinctus* and *M. spixii*. They also note that *M. surinamensis* venom was not significantly neutralized by the antivenom.

Table 2 shows the existence of a large range of both qualitative and quantitative variations in *Micrurus* venoms, probably reflecting the adaptation of the snakes from this genus, to vastly dissimilar habitats. Thus, the comparative analysis of distinct phenotypes, particularly the venom constituents and their toxic activities, reveals the heterogeneous complexity of the *Micrurus* venoms ascertaining that both the structural and the ecological evolutions constrain specific characters for adaptive values. The most striking example is given by *M. surinamensis*, a snake that inhabits an extremely distinct environment, whose venom expresses limited composition. Besides, it was showed that the antivenom used for human therapy in Brazil is not fully able to neutralize the main toxic activities present in all *Micrurus* spp venoms, indicating that, for the preparation of the Brazilian coral snake antivenom, other venoms should be included in the immunization mixture.

Taking into account the decision made by PAHO/WHO [46] and the countries of the Americas to promote strategies to diminish the health burden of accidents involving poisonous animals in the countries of Latin America, it would be lawful to consider the possibility to prepare a continental coral snake antivenom, thus contributing to countries where national production is insufficient or where it does not have manufacturing laboratories. The appropriate cooperation by scientists in various countries in order to prepare multivalent coral snake antivenom has already been proposed by Bolaños et al. [46], as early as the 1970's, but until now this relevant aim for the public health of the Americas has not been achieved. Data present in the literature, and results obtained in this study, should encourage PAHO to coordinate a regional cooperative effort to produce multivalent continental *Micrurus* antivenom that would have an important impact in the treatment of accidents involving coral snakes over the entire continent.
